# Genetic polymorphism of the Dab2 gene and its association with Type 2 Diabetes Mellitus in the Chinese Uyghur population

**DOI:** 10.7717/peerj.15536

**Published:** 2023-06-21

**Authors:** Yan-Peng Li, Dilare Adi, Ying-Hong Wang, Yong-Tao Wang, Xiao-Lei Li, Zhen-Yan Fu, Fen Liu, Aibibanmu Aizezi, Jialin Abuzhalihan, Min-Tao Gai, Xiang Ma, Xiao-mei Li, Xiang Xie, Yi-Tong Ma

**Affiliations:** 1 State Key Laboratory of Pathogenesis, Prevention and Treatment of High Incidence Diseases in Central Asia, Department of Cardiology, First Affiliated Hospital of Xinjiang Medical University, Urumqi, Xinjiang, China; 2 Xinjiang Key Laboratory of Cardiovascular Disease, Clinical Medical Research Institute, First Affiliated Hospital of Xinjiang Medical University, Urumqi, Xinjiang, China; 3 Center of Health Management, First Affiliated Hospital of Xinjiang Medical University, Urumqi, Xinjiang, China

**Keywords:** Human Disabled-2 (Dab2), Diabetes mellitus, Gene polymorphism

## Abstract

**Objective:**

The human Disabled-2 (Dab2) protein is an endocytic adaptor protein, which plays an essential role in endocytosis of transmembrane cargo, including low-density lipoprotein cholesterol (LDL-C). As a candidate gene for dyslipidemia, Dab2 is also involved in the development of type 2 diabetes mellitus(T2DM). The aim of this study was to investigate the effects of genetic variants of the Dab2 gene on the related risk of T2DM in the Uygur and Han populations of Xinjiang, China.

**Methods:**

A total of 2,157 age- and sex-matched individuals (528 T2DM patients and 1,629 controls) were included in this case-control study. Four high frequency SNPs (rs1050903, rs2255280, rs2855512 and rs11959928) of the Dab2 gene were genotyped using an improved multiplex ligation detection reaction (iMLDR) genotyping assay, and the forecast value of the SNP for T2DM was assessed by statistical analysis of clinical data profiles and gene frequencies.

**Results:**

We found that in the Uygur population studied, for both rs2255280 and rs2855512, there were significant differences in the distribution of genotypes (AA/CA/CC), and the recessive model (CC *vs.* CA + AA) between T2DM patients and the controls (*P* < 0.05). After adjusting for confounders, the recessive model (CC *vs.* CA + AA) of both rs2255280 and rs2855512 remained significantly associated with T2DM in this population (rs2255280: OR = 5.303, 95% CI [1.236 to −22.755], *P* = 0.025; rs2855512: OR = 4.892, 95% CI [1.136 to −21.013], *P* = 0.033). The genotypes (AA/CA/CC) and recessive models (CC *vs.* CA + AA) of rs2855512 and rs2255280 were also associated with the plasma glucose and HbA1c levels (all *P* < 0.05) in this population. There were no significant differences in genotypes, all genetic models, or allele frequencies between the T2DM and control group in the Han population group (all *P* > 0.05).

**Conclusions:**

The present study suggests that the variation of the Dab2 gene loci rs2255280 and rs2855512 is related to the incidence of T2DM in the Uygur population, but not in the Han population. In this study, these variations in Dab2 were an independent predictor for T2DM in the Uygur population of Xinjiang, China.

## Introduction

Type 2 diabetes mellitus (T2DM) is the most common chronic metabolic disease and has become a significant health concern because of its global impact on public health and economic development (Lin et al., 2020; Magliano et al., 2019). In China, the health burden of T2DM and its complications is increasing because of: an increased T2DM prevalence; low and stagnated rates of awareness, treatment, and disease control; high overweight and obesity rates; and worsened reported lifestyle factors (Wang et al., 2021). T2DM is a severe metabolic disorder marked by insulin resistance resulting in hyperglycemia accompanied by several comorbidities, including cardiovascular (CVD), liver, and neurological diseases (McCrimmon, Ryan & Frier, 2012). T2DM is usually caused by metabolic, environmental, behavioral, and genetic risk factors, with genetic factors accounting for a considerable proportion of cases (Kim & Egan, 2008).

The human Disabled-2 (Dab2) gene is located on chromosome 5p13 (Ogbu et al., 2021) and encodes a protein with molecular weight of 96 kDa (Xie et al., 2014). The Dab2 protein, also named DOC-2, is a putative tumor suppressor initially identified by Gertler et al. (1989) in ovarian carcinomas. Functionally Dab2 is involved in a wide variety of signaling pathways and plays an integral role in endocytosis, modulating immune function and platelet function (Nie et al., 2021). Dab2 is also involved in the regulation of blood lipid, and blood glucose metabolism. Maurer & Cooper (2006a) first reported that Dab2, as a cargo-specific endocytic adaptor protein, mediates endocytosis of LDLR on parallel pathways with autosomal recessive hypercholesterolemia protein (ARH)/adaptor protein-2 (AP-2), ultimately leading to a reduction in plasma LDL-C levels. Wei et al. (2014) further studied the mechanism of Dab2 and involvement in the regulation of lipid metabolism. Previous studies, including genome-wide association studies (GWAS) and mendelian randomization (MR) analyses, have demonstrated the causal relationship between plasma LDL-C levels and T2DM liability (Fall et al., 2015; Wei et al., 2021). Increased genetically proxied LDL-C levels were associated with increased T2DM liability in individuals with African ancestry, (Soremekun et al., 2022) while a genetically-induced elevation of circulated LDL-C levels was associated with a lower risk of T2DM in individuals with European or Asian ancestry (Hsu et al., 2022; White et al., 2016). The cause–effect relationship between LDL-C levels and T2DM has been suggested to be both population and mechanism-specific (Goff et al., 2020). Some evidence suggests that an increased risk of T2DM is a consequence of the inhibition of 3-hydroxy-3-methylglutaryl-CoA reductase (HMGCR) (Swerdlow et al., 2015), while other studies have shown the possibility of diabetogenic, LDL-C-increasing pathways, acting independently of HMGCR (Soremekun et al., 2022). In our previous study, we found an association between genetic polymorphism of Dab2 and an increased risk of coronary artery disease in the Chinese Han population (Wang et al., 2020). However, at the population level, the relationship between Dab2 gene polymorphism and T2DM has not been extensively studied (Corbi et al., 2020; Meigs et al., 2007). In this study, we aimed to explore the predictive value of Dab2 gene polymorphisms on the risk of T2DM in different population groups.

## Materials & Methods

### Human subjects and biochemical analysis

The Ethics Committee of the first affiliated hospital of Xinjiang Medical University (Urumqi, China) approved this retrospective study (20190505-01), and informed consent was obtained from each study participant. We used PASS 15.0.5 software to calculate that the minimum sample size needed for this study was 489 (Power ≥ 0.9, *α* < 0.05) (Xu et al., 2013a). We randomly recruited 2,500 inpatients of the heart center of the first affiliated hospital of Xinjiang Medical University from September 1, 2016 to December 31, 2021. A total of 2,157 subjects were included (528 T2DM, 1,629 control) in the final case-control study; among them, 1,326 subjects were Han Chinese (331 T2DM, 995 control) and 831 subjects were Uyghur Chinese (197 T2DM, 634 control). T2DM was identified using discharge diagnoses obtained from the electronic medical records of the patients, and any patient with a new or existing T2DM diagnosis was included in the T2DM group. The inclusion criteria of the T2DM group were: subjects with a fasting plasma glucose (FBS) ≥ 126mg/dL ( ≥7.0 mmol/L) or two-hour post prandial blood sugar (PPBS) concentrations ≥ 200 mg/ dL (≥11.1 mmol/L) in the oral glucose tolerance test (Joseph et al., 2022), or subjects who had previously used anti-diabetic drugs (American Diabetes Association Professional Practice C, 2022). Control subjects also received related laboratory testing to verify they did not have T2DM. Subjects were not included in the study if they had been diagnosed with: type 1 diabetes, gestational diabetes, or secondary diabetes; any severe liver, kidney, or hematopoietic system disease; or thyroid dysfunction. A standard venipuncture technique was used to obtain blood samples from the participants. Complete blood testing and biochemical assays were performed using the equipment for chemical analysis in the clinical laboratory department of the First Affiliated Hospital of Xinjiang Medical University. We extracted the DNA from the blood leukocytes at the periphery using the blood genome extraction kit (Adi et al., 2021). BMI was obtained by calculating the participant’s weight divided by the square of their height (kg/m^2^). We defined hypertension as a systolic blood pressure ≥140 mmHg and/or a diastolic blood pressure ≥90 mmHg in at least two clinic blood pressure measurements on different days (Kollias et al., 2022). We defined smoking as currently or previously smoking, and drinking was defined as drinking alcohol at least once a week for at least a year (Xie et al., 2010).

### Genotyping

**Figure 1:**
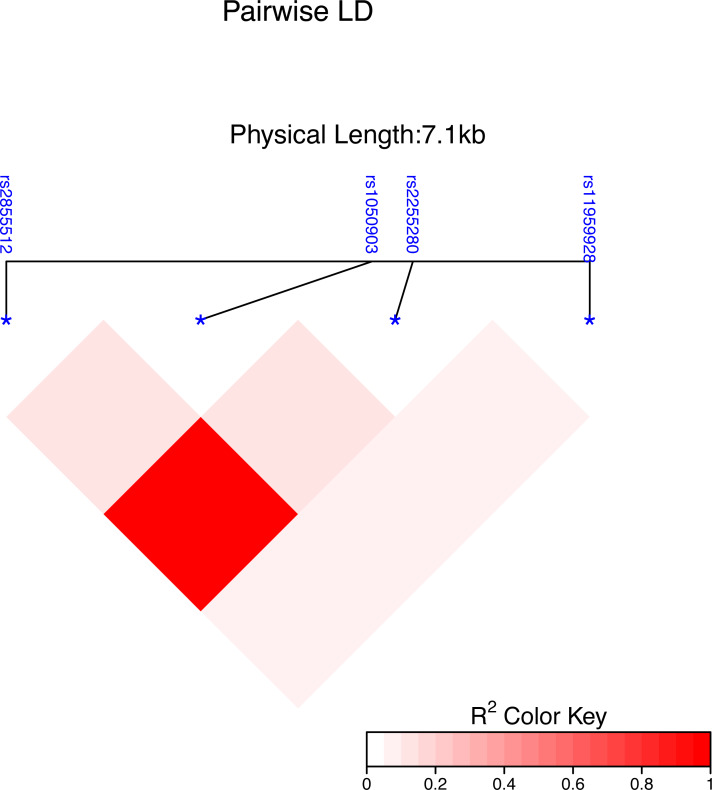
Genetic variation of the human Dab2 gene. SNP heat map of four genotyped SNPs in the study subjects, using the R package “LD heat map. ” LD blocks were identified using the solid spline and not solid spline method in the R 4.2.1 software. The LD values are displayed as follows: R^2^ color scheme: R^2^ = 0: white; 0 < *R*^2^ < 1: shades of pink; and R^2^ = 1: red.

Using the Haploview 4.2 software and International HapMap Project website phase II data base, we obtained four single nucleotide polymorphisms (SNPs) of the Dab2 gene: rs1050903, rs2255280, rs2855512 and rs11959928, by using minor allele frequency (MAF) ≥0.05 and linkage disequilibrium patterns with r^2^ ≥0.8 as a cutoff. The linkage disequilibrium (LD) map of these four tag SNPs is shown in Fig. 1. The genotyping of the SNPs was performed using an improved multiplex ligation detection reaction (iMLDR) technique (Genesky Biotechnologies Inc., Shanghai, China). Genotyping was performed in a blinded fashion without knowledge of the participants’ clinical data, and a total of 10% of the genotyped samples were duplicated to monitor genotyping quality.

### Statistical analysis

IBM SPSS Statistics 27.0 for Windows and R version 4.2.1 (R Core Team, 2022) were used to perform the statistical analyses in this study. We chose the double-tail *P*-value of 0.05 as the statistically significant threshold. The mean ±standard deviation (SD) represents the continuous variable under normal distribution, and the median (25th, 75th percentiles) represents the continuous variable with non-normal distribution. The difference between two groups was assessed with the two-independent-sample *T*-test or the Mann–Whitney U test. The categoric variables were described using numbers and percentages (%), and the *χ*^2^ test was used to analyze differences. The allele and genotype differences among groups were evaluated by the Hardy-Weinberg equilibrium (HWE) and an unconditional logistic regression analysis was performed to evaluate the value of Dab2 polymorphism on T2DM, according to computing crude or adjusted ORs and 95%Cls.

A nomogram was used to build a multi-factor regression model, score the value level of each influencing factor in the model according to the contribution degree (the size of regression coefficient) add all the scores to get the total score, and finally calculate the predicted value of the individual’s final event through the functional transformation relationship between the total score and the probability of the final event (Zhang et al., 2021). Nomograms are often used by clinicians to visualize the regression equation to evaluate a patient’s condition and prognosis (Balachandran et al., 2015; Lv et al., 2022) The scoring system in the nomogram in this study was generated by the Regression Modeling Strategies (RMS) package in R (version 4.2.1).

The nomogram model was evaluated from three aspects: discrimination ability, calibration ability, and clinical effectiveness. A receiver operation characteristic curve (ROC) was used to evaluate the discrimination ability. The value of the area under curve (AUC) is always between 0.5 and 1, with an AUC value closer to 1 indicating a good performance of the predictive model. The C-index is used to measure how well a model predicts disease risk, with an absolute value close to 1 indicating strong predictive ability of the model. Calibration plots assess the predictive accuracy and agreement between predicted and observed severity.

## Results

### Characteristics of study participants

The general data and clinical characteristics of the T2DM group and the control group of the Han and Uygur population included in this study are described in Tables 1 and 2. For both populations, there were no significant differences in age and sex between the T2DM group and the control group. In the Han population, there were no significant differences in body mass index (BMI) and triglyceride (TG) levels and the ratio of reported drinkers (all *P* > 0.05). The Han population T2DM group had higher levels of systolic blood pressure (SBP), HbA1c, left ventricular ejection fractions (LVEF), total cholesterol (TC), LDL-C, smoking, and a higher hypertension ratio than the control group (all *P* < 0.05, Table 1). In the Uygur population, there were no significant differences in high-density lipoprotein cholesterol (HDL-C) levels and the ratio of reported smokers and drinkers between the two groups (all *P* >  0.05), but the T2DM group had a significantly higher ratio of hypertension and significantly higher BMI, SBP, HbA1c, TG, TC, and LDL-C levels (all *P* < 0.05, Table 2) than those in the control group.

**Table 1:** Demographic and clinical characteristics of the Han population. Continuous variables are expressed as mean ± SD, or median (25th, 75th percentiles). Categorical variables are expressed as number and percentage. The *P* value of the continuous variables was calculated by the independent-sample *t*-test. The *P* value of the categorical variables was calculated by *χ*^2^ test.

Variables	Total Cohort (*n* = 1,326)	T2DM (*n* = 331)	Control (*n* = 995)	*P* value
Age (years)	60.68 ± 11.14	61.47 ± 10.92	60.42 ± 11.18	0.140
Male, *n* (%)	853 (64.3%)	221 (66.8%)	632 (63.5%)	0.290
Smoking, *n* (%)	508 (54.8%)	116 (47.9%)	392 (57.2%)	**0.013**
Drinking, *n* (%)	233 (25.4%)	53 (22.2%)	180 (26.5%)	0.196
Hypertension, *n* (%)	339 (51.9%)	125 (61.3%)	214 (47.7%)	**0.001**
BMI (kg/m^2^)	25.56 ± 3.40	25.90 ± 3.20	25.45 ± 3.45	0.194
SBP (mmHg)	142.99 ± 31.50	149.68 ± 33.16	140.77 ± 30.63	**<0.001**
DBP (mmHg)	89.32 ± 45.89	90.04 ± 19.24	89.08 ± 51.81	0.791
HbA1c, %	5.58 (5.28, 6.65)	7.84 (7.46, 8.25)	5.44 (5.19, 5.66)	**<0.001**
FBS (mmol/L)	6.03 ± 2.56	9.12 ± 2.12	4.99 ± 0.80	**<0.001**
TG (mmol/L)	1.88 ± 1.81	2.04 ± 1.35	1.83 ± 1.94	0.065
TC (mmol/L)	4.10 ± 1.08	4.22 ± 1.18	4.06 ± 1.04	**0.025**
HDL-C (mmol/L)	1.09 ± 0.43	1.04 ± 0.32	1.11 ± 0.46	**0.005**
LDL-C (mmol/L)	2.55 ± 0.87	2.69 ± 0.92	2.50 ± 0.85	**0.001**
Uric acid (µmol/L)	305.94 ± 103.71	281.62 ± 105.83	314.02 ± 101.76	**<0.001**
BUN (mmol/L)	6.28 ± 7.72	6.20 ± 5.47	6.30 ± 8.33	0.833
Cr (mmol/L)	74.37 ± 40.34	74.17 ± 38.69	74.43 ± 40.88	0.920
LVEF, %	63 (60, 66)	62 (57, 65)	63 (60, 67)	**0.002**
Diabetic complications				
CAD	–	82(24.8%)	–	
peripheral neuropathy	–	61(18.4%)	–	
diabetic retinopathy	–	51(15.4%)	–	
Diabetes Medication				
Metformin	–	178(53.8%)	–	
acarbose	–	71(21.5%)	–	
Glinides	–	33(9.97%)	–	
insulin	–	152(45.9%)	–	

**Notes.**

BMI body mass index SBP systolic blood pressure DBP diastolic blood pressure FBS fasting plasma glucose TG triglyceride TC total cholesterol HDL-C high-density lipoprotein cholesterol LDL-C low-density lipoprotein cholesterol BUN blood urea nitrogen Cr creatinine LVEF left ventricular ejection fractions CAD coronary atherosclerotic heart disease

The bold styling indicates that the P value is less than 0.05, and there is a significant difference between the case group and the control group.

**Table 2:** Demographic and clinical characteristics of the Uygur population. Continuous variables are expressed as mean ± SD, or median (25th, 75th percentiles). Categorical variables are expressed as number and percentage. The *P* value of the continuous variables was calculated by the independent-sample *t*-test. The *P* value of the categorical variables was calculated by *χ*^2^ test.

Variables	Total Cohort (*n* = 831)	T2DM (*n* = 197)	Control (*n* = 634)	*P* Value
Age (years)	56.06 ± 10.09	56.38 ± 9.17	55.96 ± 10.36	0.584
Male, *n* (%)	626 (75.3%)	144 (73.1%)	482 (76.0%)	0.397
Smoking, *n* (%)	372 (70.9%)	86 (67.2%)	286 (72.0%)	0.315
Drinking, *n* (%)	102 (19.5%)	24 (18.8%)	78 (19.8%)	0.898
Hypertension, *n* (%)	86 (42.0%)	32 (56.1%)	54 (36.5%)	**0.012**
BMI (kg/m^2^)	27.10 ± 3.98	27.81 ± 4.02	26.87 ± 3.95	**0.007**
SBP (mmHg)	143.11 ± 33.67	148.33 ± 32.23	141.48 ± 33.98	**0.039**
DBP (mmHg)	89.03 ± 21.46	90.07 ± 23.21	88.71 ± 20.91	0.524
HbA1c, %	5.41 (5.16, 6.28)	7.88 (7.37, 8.46)	5.30 (5.08, 5.52)	**<0.001**
FBS (mmol/L)	6.15 ± 2.83	10.01 ± 2.39	4.93 ± 0.85	**<0.001**
TG (mmol/L)	2.32 ± 3.29	3.13 ± 4.95	2.06 ± 2.52	**0.004**
TC (mmol/L)	4.21 ± 1.68	4.61 ± 2.34	4.08 ± 1.39	**0.003**
HDL-C (mmol/L)	1.12 ± 1.38	1.30 ± 2.20	1.06 ± 0.99	0.143
LDL-C (mmol/L)	2.54 ± 0.94	2.74 ± 1.01	2.47 ± 0.91	**<0.001**
Uric acid (µmol/L)	290.54 ± 109.73	268.08 ± 108.43	297.67 ± 109.27	**<0.001**
BUN (mmol/L)	6.67 ± 8.79	6.32 ± 6.28	6.78 ± 9.44	0.528
Cr (mmol/L)	77.49 ± 50.76	71.73 ± 41.09	79.30 ± 53.33	0.072
LVEF, %	62 (57, 65)	61 (56, 65)	62 (57, 66)	0.270
Diabetic complications				
CAD	–	38(19.3%)	–	
peripheral neuropathy	–	45(22.8%)	–	
diabetic retinopathy	–	28(14.2%)	–	
Diabetes Medication				
Metformin	–	95(48.2%)	–	
acarbose	–	40 (20.3%)	–	
Glinides	–	16(8.1%)	–	
insulin	–	83(42.1%)	–	

**Notes.**

BMI body mass index SBP systolic blood pressure DBP diastolic blood pressure FBS fasting plasma glucose TG triglyceride TC total cholesterol HDL-C high-density lipoprotein cholesterol LDL-C low-density lipoprotein cholesterol BUN blood urea nitrogen Cr creatinine LVEF left ventricular ejection fractions CAD coronary atherosclerotic heart disease

The bold styling indicates that the P value is less than 0.05, and there is a significant difference between the case group and the control group.

### The genotype and allele distributions of the selected SNPs in the T2DM group and controls

The genotype distributions of the four SNPs were under the Hardy-Weinberg equilibrium (all *P* > 0.05) in both the T2DM and the control groups. However, there may be significant differences in gene variations when considering the genetic backgrounds of different populations. To test this possibility, we compared the distributions of genotype and allele frequency between the two study populations (Table S1). The distribution of the genotypes, genetic models and allele frequency all showed significant differences between the Han and Uygur populations for rs2255280, rs2855512, and rs11959982 (all *P* < 0.001). Based on these results showing the impact of population stratification, we divided the subjects into two groups, Han and Uyghur Chinese population group, and all analyses were performed separately.

Table 3 shows the distribution of genotypes, genetic models (dominant model, recessive model, and overdominant model) (Sabourin, Nobel & Valdar, 2015) and allele frequencies for the four SNPs (rs1050903, rs2255280, rs2855512, and rs11959982) of the Dab2 gene in the Han population group. There were no significant differences in genotypes, all genetic models, or allele frequencies between the T2DM and control group in the Han population group (all *P* > 0.05, Table 3).

**Table 3:** Genotype and allele distribution of SNPs of the Dab2 gene in the Han population.

SNPs			T2DM, *n* (%)	Control, *n* (%)	*P* value
rs1050903	Genotype	GG	192 (58.71%)	538 (54.50%)	0.384
(G >C)		GC	117 (35.77%)	395 (40.02%)	
		CC	18 (5.50%)	54 (5.47%)	
	Dominant model	GG	192 (58.72%)	538 (54.51%)	0.185
		GC+CC	135 (41.28%)	449 (45.49%)	
	Recessive model	CC	18 (5.51%)	54 (5.47%)	0.982
		GG+GC	309 (94.49%)	933 (94.53%)	
	Overdominant model	GC	117 (35.78%)	395 (40.02%)	0.173
		GG+CC	210 (64.22%)	592 (59.98%)	
	Allele	G	501 (76.6%)	1471 (74.5%)	0.285
		C	153 (23.4%)	503 (25.5%)	
rs2255280	Genotype	AA	147 (45.09%)	427 (43.30%)	0.754
(A >C)		CA	145 (44.47%)	462 (46.85%)	
		CC	34 (10.42%)	97 (9.83%)	
	Dominant model	AA	147 (45.09%)	427 (43.31%)	0.573
		CA+CC	179 (54.91%)	559 (56.69%)	
	Recessive model	CC	34 (10.43%)	97 (9.84%)	0.757
		AA+CA	292 (89.57%)	889 (90.16%)	
	Overdominant model	CA	145 (44.48%)	462 (46.86%)	0.455
		AA+CC	181 (55.52%)	524 (53.14%)	
	Allele	A	439 (67.3%)	1316 (66.7%)	0.779
		C	213 (32.7%)	656 (33.3%)	
rs2855512	Genotype	AA	148 (45.26%)	425 (43.06%)	0.721
(A >C)		CA	145 (44.35%)	463 (46.91%)	
		CC	34 (10.39%)	99 (10.03%)	
	Dominant model	AA	148 (45.26%)	425 (43.06%)	0.487
		CA+CC	179 (54.74%)	562 (56.94%)	
	Recessive model	CC	34 (10.40%)	99 (10.03%)	0.848
		AA+CA	293 (89.60%)	888 (89.97%)	
	Overdominant model	CA	145 (44.34%)	463 (46.91%)	0.420
		AA+CC	182 (55.66%)	524 (53.09%)	
	Allele	A	441 (67.48%)	1313 (66.51%)	0.666
		C	213 (32.62%)	661 (33.49%)	
rs11959928	Genotype	TT	238 (72.78%)	737 (74.67%)	0.473
(T >A)		AT	85 (25.99%)	231 (23.40%)	
		AA	4 (1.22%)	19 (1.92%)	
	Dominant model	TT	238 (72.78%)	737 (74.67%)	0.499
		AT+AA	89 (27.22%)	250 (25.33%)	
	Recessive model	AA	4 (1.22%)	19 (1.93%)	0.402
		TT+AT	323 (98.78%)	968 (98.07%)	
	Overdominant model	AT	85 (25.99%)	231 (23.40%)	0.342
		AA+TT	242 (74.01%)	756 (76.60%)	
	Allele	T	561 (85.78%)	1705 (86.37%)	0.703
		A	93 (14.22%)	269 (13.63%)	

Table 4 shows the distribution of genotypes, genetic models, and alleles for the four SNPs (rs1050903, rs2255280, rs2855512, and rs11959982) of the Dab2 gene in the Uygur population group. For rs2255280, the distribution of the genotypes and the recessive model (CC *vs.* AA + CA) showed significant differences between the T2DM and the control group (*P* = 0.011 and *P* = 0.011, respectively). This suggests that patients with the CC genotypes of rs2255280 may have a lower risk of T2DM. For rs2855512, the distribution of the genotypes and the recessive model (CC *vs.* AA + CA) also showed significant differences between T2DM and the control group (*P* = 0.017 and *P* = 0.015), indicating that patients with the CC genotype of rs22855512 might also have a lower risk of T2DM than those with AA or CA genotypes. There were no significant differences for rs1050903 or rs11959982 in the distribution genotypes and different genetic models between the T2DM and the control group in the Uygur population group (all *P* >0.05).

**Table 4:** Genotype and allele distribution of SNPs of the Dab2 gene in the Uygur population.

SNPs			T2DM, *n* (%)	control, *n* (%)	*P* value
rs1050903	Genotype	GG	108 (55.11%)	363 (57.52%)	0.814
(G >C)		GC	77 (39.28%)	232 (36.77%)	
		CC	11 (5.61%)	36 (5.71%)	
	Dominant model	GG	108 (55.10%)	363 (57.53%)	0.549
		GC+CC	88 (44.90%)	268 (42.47%)	
	Recessive model	CC	11 (5.61%)	36 (5.71%)	0.961
		GG+GC	185 (94.39%)	595 (94.29%)	
	Overdominant model	GC	77 (39.29%)	232 (36.77%)	0.524
		GG+CC	119 (60.71%)	399 (63.23%)	
	Allele	G	293 (74.74%)	958 (75.91%)	0.638
		C	99 (25.26%)	304 (24.09%)	
rs2255280	Genotype	AA	129 (65.82%)	432 (68.47%)	**0.011**
(A >C)		CA	65 (33.16%)	166 (26.31%)	
		CC	2 (1.02%)	33 (5.22%)	
	Dominant model	AA	129 (65.82%)	432 (68.46%)	0.488
		CA+CC	67 (34.18%)	199 (31.54%)	
	Recessive model	CC	2 (1.02%)	33 (5.23%)	**0.011**
		AA+CA	194 (98.98%)	598 (94.77%)	
	Overdominant model	CA	65 (33.16%)	166 (26.31%)	0.062
		AA+CC	131 (66.84%)	465 (73.69%)	
	Allele	A	325 (82.07%)	1030 (81.61%)	0.838
		C	71 (17.93%)	232 (18.38%)	
rs2855512	Genotype	AA	129 (65.81%)	432 (68.46%)	**0.017**
(A >C)		CA	65 (33.16%)	168 (26.62%)	
		CC	2 (1.02%)	31 (4.91%)	
	Dominant model	AA	129 (65.82%)	432 (68.46%)	0.488
		CA+CC	67 (34.18%)	199 (31.54%)	
	Recessive model	CC	2 (1.02%)	31 (4.91%)	**0.015**
		AA+CA	194 (98.98%)	600 (95.09%)	
	Overdominant model	CA	65 (33.16%)	168 (26.62%)	0.075
		AA+CC	131 (66.84%)	463 (73.38%)	
	Allele	A	323 (82.40%)	1032 (81.77%)	0.779
		C	69 (17.60%)	230 (18.23%)	
rs11959928	Genotype	TT	89 (45.40%)	326 (51.66%)	0.310
(T >A)		AT	86 (43.87%)	246 (38.98%)	
		AA	21 (10.71%)	59 (9.35%)	
	Dominant model	TT	89 (45.41%)	326 (51.66%)	0.126
		AT+AA	107 (54.59%)	305 (48.34%)	
	Recessive model	AA	21 (10.71%)	59 (9.35%)	0.573
		TT+AT	175 (89.29%)	572 (90.65%)	
	Overdominant model	AT	86 (43.88%)	246 (38.99%)	0.222
		AA+TT	110 (56.12%)	385 (61.01%)	
	Allele	T	264 (67.35%)	898 (71.16%)	0.149
		A	128 (32.65%)	364 (28.84%)	

### Multivariable logistic regression analysis for T2DM and control group in the Uygur population group

Tables 5 and 6 show the multivariable logistic regression analyses of the major confounding factors for T2DM in the Uygur population group. After multivariate adjustment for the confounders, such as BMI, TG, TC, LDL-C, and prevalence of smoking and hypertension, the recessive model of rs2255280 (CC *vs.* CA+AA: OR = 5.303, 95% CI = 1.236–22.755, *P* = 0.025, Table 5) and the recessive model of rs2855512 (CC *vs.* CA+AA: OR = 4.892, 95% CI = 1.136–21.013, *P* = 0.033, Table 6) remained significantly associated with T2DM.

**Table 5:** Multivariable logistic regression analysis for T2DM and control group in the Uygur population (rs2255280).

	OR	95%CI	*P* value
rs2255280 (CC *vs.* CA+AA)	5.303	1.236–22.755	**0.025**
BMI	1.053	1.007–1.102	**0.025**
hypertension	2.174	1.546–3.058	**<0.001**
uric acid	0.997	0.995–0.999	**<0.001**
TG	1.178	1.065–1.303	**0.001**
TC	0.754	0.595–0.956	**0.020**
LDL-C	1.832	1.349–2.487	**<0.001**

**Notes.**

OR odds ratio CI confidence interval BMI body mass index TG triglyceride TC total cholesterol LDL-C low-density lipoprotein cholesterol

**Table 6:** Multivariable logistic regression analysis for T2DM and control group in the Uygur population (rs2255512).

	OR	95%CI	*P* value
rs2855512 (CC *vs.* CA+AA)	4.892	1.136–21.013	**0.033**
BMI	1.053	1.006–1.101	0.025
hypertension	2.159	1.536–3.037	**<0.001**
uric acid	0.997	0.995–0.999	**<0.001**
TG	1.180	1.066–1.305	**0.001**
TC	0.752	0.593–0.954	**0.019**
LDLC	1.841	1.356–2.500	**<0.001**

**Notes.**

OR odds ratio CI confidence interval BMI body mass index TG triglyceride TC total cholesterol LDL-C low-density lipoprotein cholesterol

### Genotypes and glucose levels

**Figure 2:**
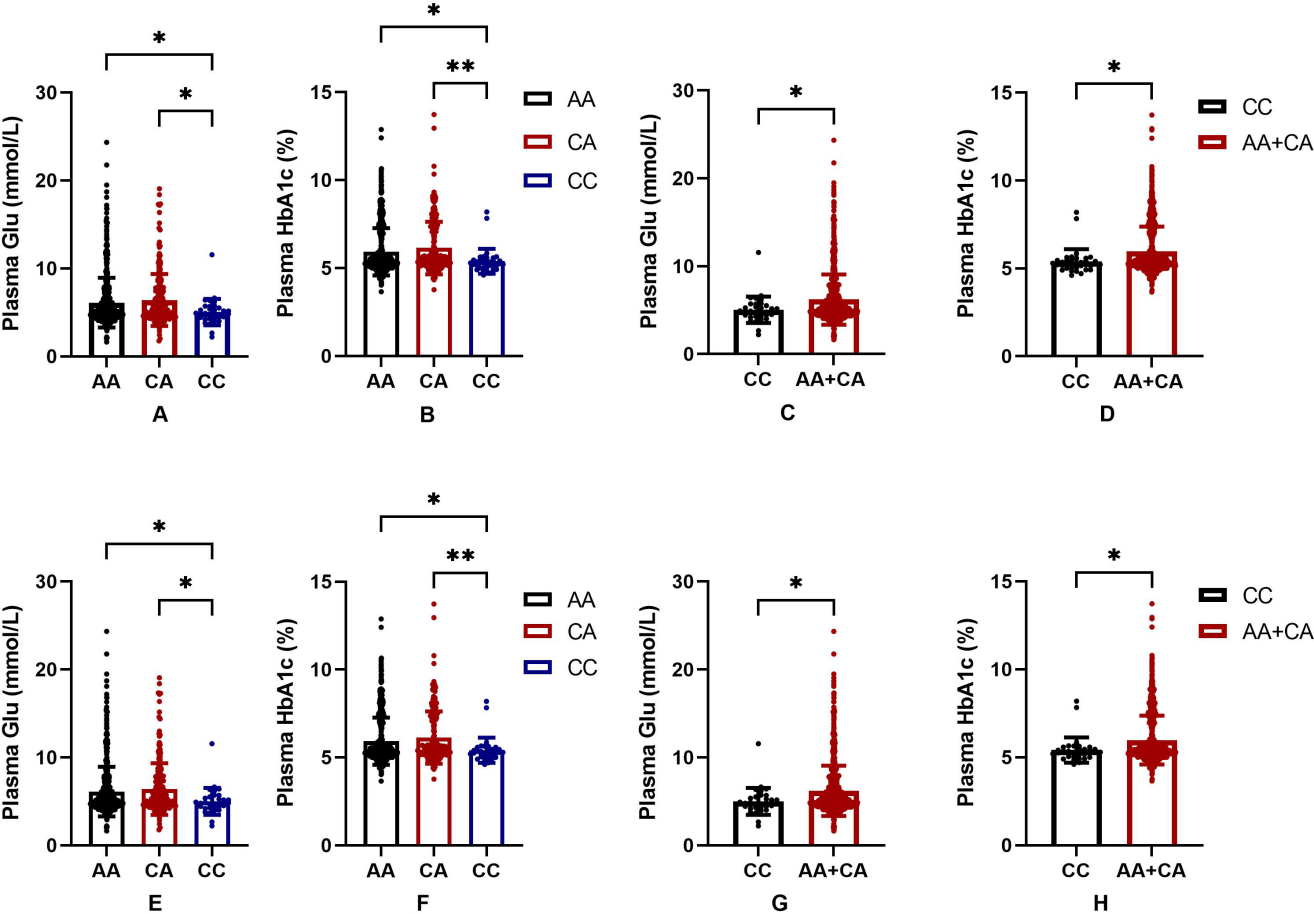
Association between SNPs and blood glucose parameters. (A–D) Influence of the Dab2 gene rs2255280 on blood glucose level; *n* = 831; (E–H) influence of the Dab2 gene rs2855512 on the blood glucose profile; *n* = 831; A, B, E, F are grouped by genotypes; C, D, G, H are grouped by the recessive model (CC *vs.* CA + AA). Values are means ± SD, *P < 0.05, **P < 0.01.

In order to further judge the relationship between SNPs and blood glucose level in the Uygur population group, we drew a bar chart of the correlation between rs2255280 and rs2855512 genotype and blood glucose level including 831 Uyghur Chinese patients (197 T2DM, 634 control). Blood glucose level was represented by FBG and HbA1c. Figure 2 shows that the plasma glucose and HbA1c levels were significantly lower in the CC genotype than that in AA or CA genotypes both in rs2255280 (Figs. 2A, 2B) and rs2855512 (Figs. 2A, 2B); *P* < 0.05). The plasma glucose and HbA1c levels were significantly lower in the recessive model (CC) than that in the AA+CA model both in rs2255280 (Figs. 2C, 2D) and rs2855512 (Figs. 2B, 2D; *P* < 0.05). After excluding 98 patients who had received drug treatment, the correlation between blood glucose level and gene polymorphism was analyzed in the remaining 733 untreated Uygur subjects (Fig. S1). Even with the possible effects of drugs on blood glucose levels excluded, the plasma glucose and HbA1c levels were still significantly lower in the recessive model (CC) than in the AA+CA model in rs2255280 (*P* < 0.05).

### Predictive nomogram for T2DM and validation of the nomogram

**Figure 3:**
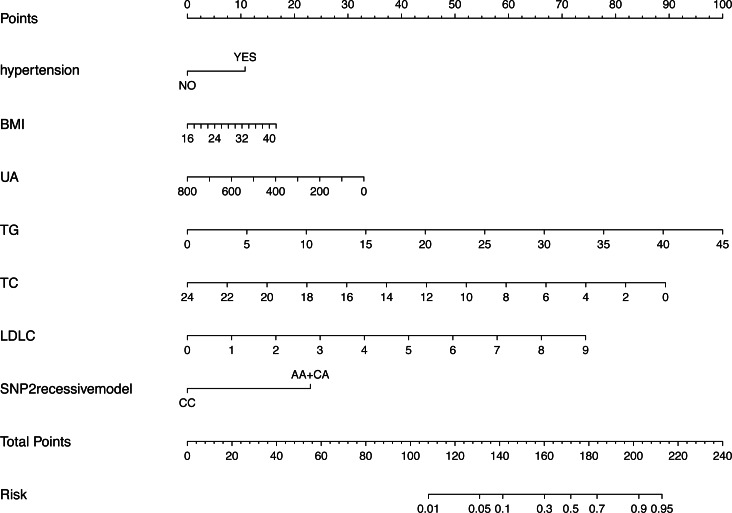
Nomogram predicting the risk of T2DM for patients with significantly different SNPs of the Dab2 gene. The value of each variable was given a score on the point scale axis. Total score is calculated by adding every individual score together and this total score is then used to estimate the probability of T2DM. Abbreviations: BMI, body mass index; UA, uric acid; TG, triglyceride; TC, total cholesterol; LDLC, low density lipoprotein cholesterol; SNP2, rs2255280; SNP3, rs2855512.

Figure 3 shows the nomogram based on the results of the logistic regression analysis. Hypertension, BMI, TG, TC, LDL-C, uric acid and rs2255280 (all *P* < 0.05) were included in the final model to develop the nomogram. The nomogram is a graphic depiction of the model, predicting the risk of T2DM. Refer to the nomogram legends for how to predict risk. For example, a person with hypertension (9.6 points), BMI of 27 (7.1 points), uric acid of 250 mmol/L (30 points), TG of 1.5 mmol/L (3.1 points), TC of 5.2 mmol/L (72.5 points), LDL-C of 2.3 mmol/L (20 points) and rs2255280 AA genotype (21 points), would have a nomogram total point score of 163.3, indicating an estimated 42.1% chance of experiencing T2DM.

**Figure 4:**
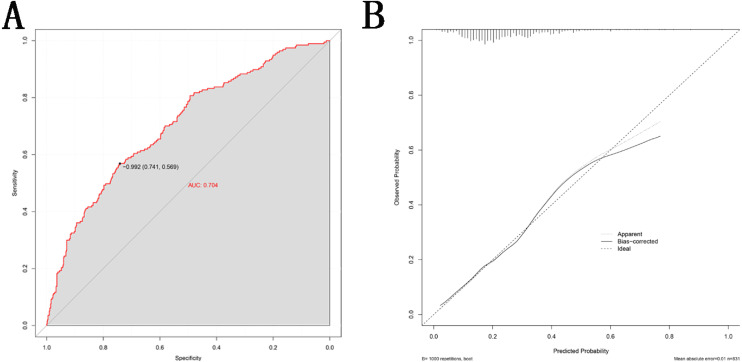
Validity of the nomogram. (A) Receiver operation characteristic curve (ROC) for validating the discrimination power of the nomogram. (B) Calibration plot of the nomogram. The diagonal line represents a perfect prediction by an ideal model. The black line represents the performance of the nomogram, of which a closer fit to the diagonal red line represents a better prediction.

This nomogram was validated internally, based on discrimination and calibration, using the bootstrap method with 1,000 resamples. As shown in Fig. 4A, the AUC value of the nomogram was 0.704 (95% CI [0.662–0.746]; *P* < 0.001) and the C-index was 0.704 (95% CI [0.663–0.776]; *P* < 0.001), indicating the model had a good predictive power. The calibration curve of the model is shown in Fig. 4B.

## Discussion

In our study, we found that genetic polymorphisms of rs2255280 and rs2855512 are significantly associated with T2DM susceptibility in the Chinese Uygur population. This is the first study to explore genetic polymorphism of the Dab2 gene and the risk of T2DM (Ogbu et al., 2021).

T2DM is a complex glucose and lipid metabolism-associated disease mainly characterized by hyperglycemia arising from insulin resistance and/or insufficient insulin secretion (Chatterjee, Khunti & Davies, 2017). Previous studies (Hoenig & Sellke, 2010; Pihlajamäki et al., 2004; Taskinen & Borén, 2015) have shown that glucose and lipid metabolic disorders (GLMD) are the critical mechanisms in the pathophysiology of T2MD (Chung et al., 2020; Morigny et al., 2021). Several candidate-gene analyses have shown that the incidence of T2DM is related to abnormal candidate genes associated with glycolipid metabolism at multiple gene loci (Seino, Fukushima & Yabe, 2010).

The Dab2 gene encodes a protein consisting of 770 amino acids (Xu et al., 1995). Dab2 belongs to the clathrin-associated sorting proteins (CLASPs) family (Maurer & Cooper, 2006b). Previous studies have confirmed the essential role of the Dab2 gene in lipid metabolism (Garcia et al., 2001; Tao et al., 2016a; Tao et al., 2016b; Tao et al., 2016c). In addition to participating in lipid metabolism, (Wang, Fang & Shyu, 2018) found that the Dab2 gene is also involved in in glucose metabolism in rat cardiomyocyte models. They found that hyperglycemia increased Dab2 expression in cardiomyocytes. In contrast, inhibition of Dab2 mRNA and protein expression can reverse hyperglycemia (Besseling et al., 2015; Fall et al., 2015; Swerdlow et al., 2015). (Lu et al., 2018) further demonstrated that Dab2 functions as a negative regulator of canonical Wnt signaling by stabilizing the beta-catenin degradation complex, which may contribute to its role in regulating blood glucose levels in mice.

Dab2 gene polymorphism has been widely studied, and has found to be associated with some tumor diseases. For example, Dab2 rs2255280 is associated with a lower risk of pancreatic cancer (Wang et al., 2017), whereas Dab2 rs2243421 is a significant predictor of gastric cancer mortality (Xu et al., 2013b). Our team previously demonstrated the correlation between Dab2 and the risk of coronary artery disease in the Chinese Han population (Wang et al., 2020), and found that the rs2855512 and rs2255280 polymorphisms of Dab2 were significantly associated with an increased risk of CAD. Some previous studies have confirmed the relationship between the Dab2 gene and T2DM. In 2003, (Tanaka et al., 2003) published the first GWAS of diabetic nephropathy in a Japanese population. They found that SNPs in Dab2 were associated with diabetic nephropathy. James et al. used the Affymetrix 100K SNP array in 1,087 Framingham Offspring Study family members to test associations of SNP genotypes with incident diabetes (Meigs et al., 2007). They found 128 SNPs, some of which are located at the Dab2 gene locus, that were associated with incident diabetes. (Corbi et al., 2020) identified that Dab2 was one of the differentially expressed genes (DEGs) of circulating lymphocytes and monocytes in T2MD, however, more evidence is needed to verify this conclusion.

Our study provides evidence of a significant correlation between Dab2 gene polymorphism and an elevated risk of T2DM usng statistical analysis. Our findings are based on the fact that there were only two patients with a CC genotype of rs2255280 in the T2DM group *vs.* 33 in the control group. Though it may seem that these small numbers might affect the statistical power of the findings, that was not the case in this study. To verify our findings, we used the NCBI databases to obtain the aggregate allele frequency of rs2255280 from dbGaP provided by ALFA project: *C* = 0.002964 (European); *C* = 0.0029 (African); *C* = 0.0030 (African American); *C* = 0.3265 (Asian); *C* = 0.3324 (East Asian); *C* = 0.1206 (Latin American). Among the 827 study participants in the Uyghur population group, 35 (0.04%) had CC genotypes, while 131 (9.87%) of the 1,326 total study participants in the Han population group had a CC genotype. Based on the ALFA project data for rs2255280 in European and Asian populations, the mutation frequency found in our study is not lower than expected. Considering that only two cases in the T2DM group had CC genotypes, in order not to affect the statistical efficiency, we used the Fisher exact probability method to detect the difference in chi-square test. We will also continue to collect samples of related metabolic diseases for expanding sample size verification and correlation analyses for future studies.

We found that the variation of the Dab2 gene loci rs2255280 and rs2855512 is associated with the incidence of T2DM in the Uygur Chinese population group. However, the same association was not observed in the Han population group. There are several possible reasons for these results. First, diabetes is a hereditary disease, and different races have different susceptibility and prevalence rates due to different genetic backgrounds (Bellary et al., 2021). Yang et al. (2012) reported that the prevalence of diabetes was 6.23% in the Uygur population and 9.26% in the Han population in 2012. Secondly, the traditional diet of the Uyghur population is based mainly on pasta, beef, mutton and dairy products, while the traditional diet of the Han population is mainly vegetables and rice, leading to population differences in glucose and lipid intake and absorption (Liu et al., 2015; Tao et al., 2013). Because of different genetic backgrounds, unique eating customs and different ecological environments, the abnormal rate of blood glucose and lipids in different nationalities in Xinjiang is higher than the national average. Therefore, the predictive value of different polymorphisms of the Dab2 gene on the risk of diabetes differs in different populations (Chen et al., 2021).

We established an early warning model incorporating clinical characteristics and Dab2 gene variation that may be useful as a predictive method to further stratify the incidence risk of T2DM patients. Despite the promising findings in the present study, this study also has several limitations which should be acknowledged. This study is a single-center, retrospective study with a small sample size. Therefore, selection bias cannot be excluded. This is also an observational study that does not explore causality, so only an association between the Dab2 gene and T2DM mellitus was established. To conclude that the Dab2 gene predisposes a patient to T2DM, a large prospective, blinded study should be conducted. This study only found the correlation between SNP and T2DM, and did not verify whether SNP affects the occurrence of T2DM by affecting the expression of the Dab2 gene. Therefore, we plan to conduct further gene expression studies to judge the correlation between the expression level of serum Dab2 protein and blood glucose parameters.

## Conclusions

This study revealed that the variation of the Dab2 gene loci rs2255280 and rs2855512 is associated with the incidence of T2DM in the Uygur Chinese population. In this population, Dab2 gene polymorphism may be independently related to blood glucose metabolism.

##  Supplemental Information

10.7717/peerj.15536/supp-1 Supplemental Information 1 Raw data

10.7717/peerj.15536/supp-2 Supplemental Information 2 Association between SNPs and blood glucose parameters in untreated Uygur population (A) Influence of the Dab2 gene rs2255280 on blood glucose level in untreated Uygur population, *n* = 733; (B) Influence of the Dab2 gene rs2855512 on the blood glucose profile in untreated Uygur population, *n* = 733 ; (a), (b) are grouped by genotypes; (c), (d) are grouped by the recessive model (CC *vs.* CA + AA). Values are means ± SD, *P < 0.05, **P < 0.01.

10.7717/peerj.15536/supp-3 Supplemental Information 3 Genotype and allele distribution of SNPs of the Dab2 gene between the two population

## Additional Information and Declarations

### Data Availability

The following information was supplied regarding data availability: The raw measurements are available in the Supplemental Files.
