# Supplementary material for: Genetic polymorphism of the Dab2 gene and its association with Type 2 Diabetes Mellitus in the Chinese Uyghur population
Source: PeerJ. 2023 Jun 21;11:e15536. doi: 10.7717/peerj.15536 (PMC10290452; doi:10.7717/peerj.15536)
Supplement: Supplemental Information 3 [file peerj-11-15536-s003.docx]

Table S1 Genotype and allele distribution of SNPs of the Dab2 gene between the two population

| SNPs |  |  | Han, n (%) | Uygur, n (%) | *P* value |
| --- | --- | --- | --- | --- | --- |
| rs1050903 | Genotype | GG | 730 (55.6%) | 471 (57.0%) | 0.758 |
| (G＞C) |  | GC | 512 (39.0%) | 309 (37.4%) |  |
|  |  | CC | 72 (5.5%) | 47 (5. 7%) |  |
|  | Dominant model | GG | 730 (55.6%) | 471 (57.0%) | 0.526 |
|  |  | GC+CC | 584 (44.4%) | 356 (43.0%) |  |
|  | Recessive model | CC | 72 (5.5%) | 47 (5.7%) | 0.841 |
|  |  | GG+GC | 1242 (94.5%) | 780 (94.3%) |  |
|  | Additive model | GC | 512 (39.0%) | 309 (37.4%) | 0.458 |
|  |  | GG+CC | 802 (61.0%) | 508 (62.6%) |  |
|  | Allele | G | 1972 (75.0%) | 1251 (75.6%) | 0.659 |
|  |  | C | 656 (25.0%) | 403 (24.4%) |  |
| rs2255280 | Genotype | AA | 574 (43.8%) | 561 (67.8%) | **＜0.001** |
| (A＞C) |  | CA | 607 (46.3%) | 231 (27.9%) |  |
|  |  | CC | 131 (10.0%) | 35 (4.2%) |  |
|  | Dominant model | AA | 574(43.8%) | 561 (67.8%) | **＜0.001** |
|  |  | CA+CC | 738 (56.3%) | 266 (32.2%) |  |
|  | Recessive model | CC | 131 (10.0%) | 35 (4.2%) | **＜0.001** |
|  |  | AA+CA | 1181 (90.0%) | 792 (95.8%) |  |
|  | Additive model | CA | 607 (46.3%) | 231 (27.9%) | **＜0.001** |
|  |  | AA+CC | 705 (53.7%) | 596 (72.1%) |  |
|  | Allele | A | 1755 (66.9%) | 1353 (81.8%) | **＜0.001** |
|  |  | C | 869 (33.1%) | 301 (18.2%) |  |
| rs2855512 | Genotype | AA | 573 (43.6%) | 561 (67.8%) | **＜0.001** |
| (A＞C) |  | CA | 608 (46.3%) | 233 (28.2%) |  |
|  |  | CC | 133 (10.1%) | 33 (4.0%) |  |
|  | Dominant model | AA | 573 (43.6%) | 561 (67.8%) | **＜0.001** |
|  |  | CA+CC | 741 (56.4%) | 266 (32.2%) |  |
|  | Recessive model | CC | 133 (10.1%) | 33 (4.0%) | **＜0.001** |
|  |  | AA+CA | 1181 (89.9%) | 794 (96.0%) |  |
|  | Additive model | CA | 608 (46.3%) | 233 (28.2%) | **＜0.001** |
|  |  | AA+CC | 706 (53.7%) | 594 (71.8%) |  |
|  | Allele | A | 1754 (66.7%) | 1355 (81.9%) | **＜0.001** |
|  |  | C | 874 (33.3%) | 299 (18.1%) |  |
| rs11959928 | Genotype | TT | 975 (74.2%) | 415 (50.2%) | **＜0.001** |
| (T＞A) |  | AT | 316 (24.0%) | 332 (40.1%) |  |
|  |  | AA | 23 (1.8%) | 80 (9.7%) |  |
|  | Dominant model | TT | 975 (74.2%) | 415 (50.2%) | **＜0.001** |
|  |  | AT+AA | 339 (25.8%) | 412 (49.8%) |  |
|  | Recessive model | AA | 23 (1.8%) | 80 (9.7%) | **＜0.001** |
|  |  | TT+AT | 1291 (98.2%) | 747 (90.3%) |  |
|  | Additive model | AT | 316 (24.0%) | 332 (40.1%) | **＜0.001** |
|  |  | AA+TT | 998 (76.0%) | 495 (59.9%) |  |
|  | Allele | T | 2266 (86.2%) | 1162 (70.3%) | **＜0.001** |
|  |  | A | 362 (13.7%) | 492 (29.7%) |  |
