# Supplementary material for: Genetic polymorphism of the Dab2 gene and its association with Type 2 Diabetes Mellitus in the Chinese Uyghur population
Source: PeerJ. 2023 Jun 21;11:e15536. doi: 10.7717/peerj.15536 (PMC10290452; doi:10.7717/peerj.15536)
Supplement: Supplemental Information 2 — (A) Influence of the Dab2 gene rs2255280 on blood glucose level in untreated Uygur population, n = 733; (B) Influence of the Dab2 gene rs2855512 on the blood glucose profile in untreated Uygur population, n = 733 ; (a), (b) are grouped by genotypes; (c), (d) are grouped by the recessive model (CC vs. CA + AA). Values are means ± SD, *P < 0.05, **P < 0.01. [file peerj-11-15536-s002.pdf]

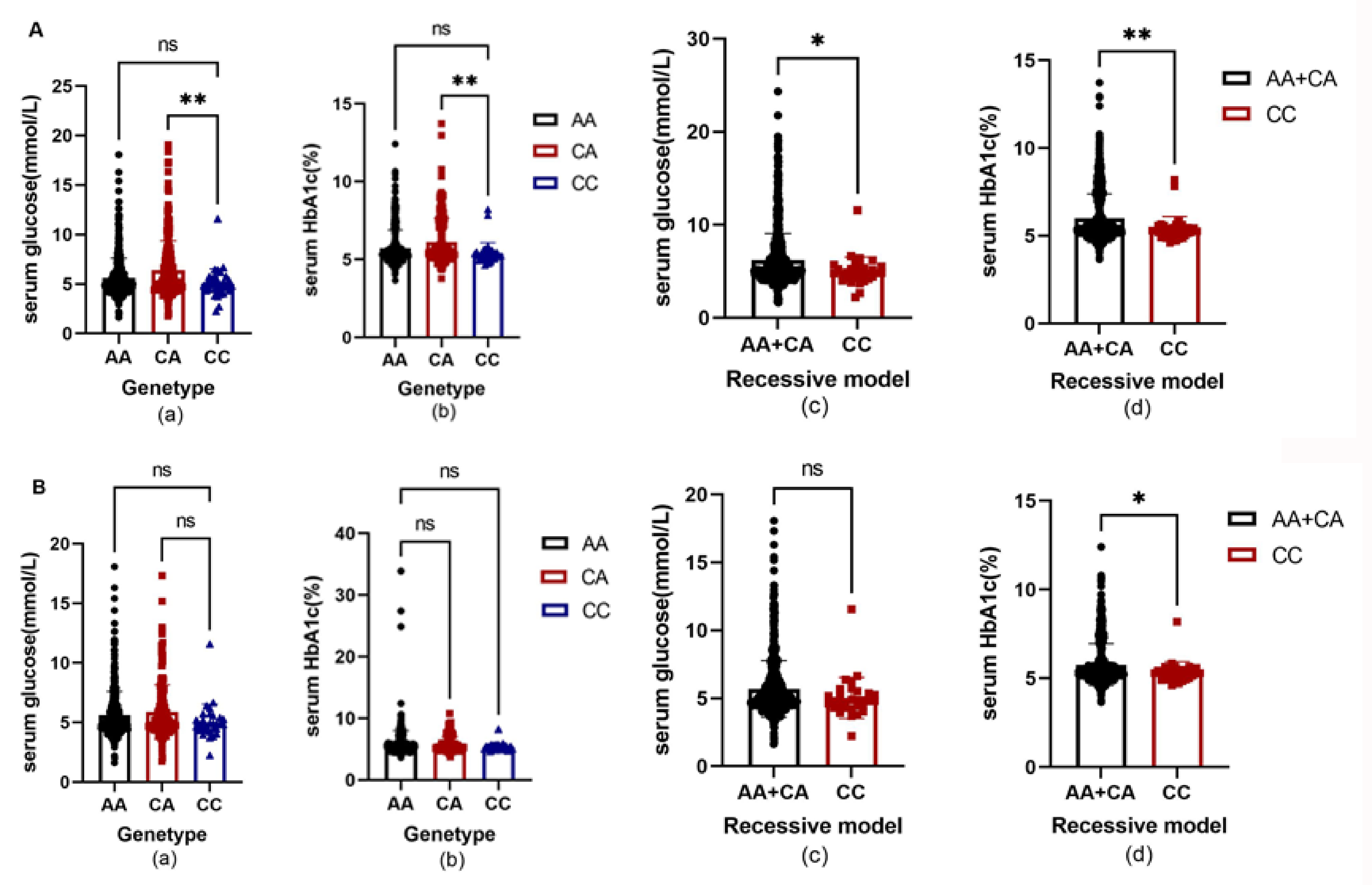
A
ns
**
serum glucose(mmol/L)
25
20
15
10
5
0
AA
CA
CC
Genetype
(a)
ns
**
serum HbA1c(%)
15
10
5
0
AA
CA
CC
Genetype
(b)
AA
CA
CC
*
serum glucose(mmol/L)
30
20
10
0
AA+CA
CC
Recessive model
(c)
**
serum HbA1c(%)
15
10
5
0
AA+CA
CC
Recessive model
(d)
AA+CA
CC
B
ns
ns
serum glucose(mmol/L)
20
15
10
5
0
AA
CA
CC
Genetype
(a)
ns
ns
serum HbA1c(%)
40
30
20
10
0
AA
CA
CC
Genetype
(b)
AA
CA
CC
ns
serum glucose(mmol/L)
20
15
10
5
0
AA+CA
CC
Recessive model
(c)
*
serum HbA1c(%)
15
10
5
0
AA+CA
CC
Recessive model
(d)
AA+CA
CC
